# The potential advantages of transplanting organs from pig to man: A transplant Surgeon's view

**DOI:** 10.4103/0970-1591.33729

**Published:** 2007

**Authors:** Carl G. Groth

**Affiliations:** Karolinska Institute, Stockholm, Sweden

**Keywords:** Pig-to-man organ transplantation, the immune barrier, risk of transmitting microorganisms, ethical issues

## Abstract

Once pig organs can be transplanted into humans, transplantation will move into a new era. There will be unlimited access to undamaged organs and cells for transplantation and, eventually, donation from deceased or live human beings will become obsolete. Furthermore, it will be possible to alleviate graft rejection, at least in part, by genetic modification of the source animal. Currently, there are three major obstacles to performing transplantations from pig to man: 1) a powerful immune barrier, 2) a potential risk of transmitting microorganisms, particularly endogenous retrovirus and 3) ethical issues related to the future recipients and to society at large.

This article will first discuss ongoing work with regards to overcoming the current obstacles. Then, the many potential advantages of using pig organs will be listed. Next, the criteria for selecting the first patients for transplantation with pig organs, will be briefly discussed. Finally, some promising observations made in the context of early attempts at transplanting porcine cells to patients, will be mentioned.

The possibility of transplanting organs and tissues between different species (xenotransplantation) has long been an elusive goal for transplant researchers. For clinicians there has been the hope that animal organs could one day be transplanted into humans. In the 1970s and 1990s, a number of attempts were made to transplant kidneys and in some instances livers and hearts, from non-human primates to patients. Some such kidneys functioned for several months, but the majority of the primate organs failed because of rejection or surgical complications, early after transplantation.[1]

In the early 1990s, there was a resurgence in interest in xenotransplantation. However, at this time primates were disfavored as the source animals. The reasons for this included a high risk of virus transmission and that most of the larger primates were classed as endangered species. Instead, the pig was, for several reasons, seen as the animal of choice. Pig organs are anatomically similar to human organs and pigs come in all sizes. Furthermore, pigs have large litters and are easy to breed. Since millions of pigs are slaughtered annually for human consumption, there could be no ethical objection to using pigs' organs for treating human disease. There was also the fact that the pig was suitable for genetic engineering.

The future utilization of pig organs and cells for transplant into humans will revolutionize transplantation. There will be unlimited access to undamaged organs and cells for transplantation and, eventually the use of human organs, from living or diseased donors, will become obsolete. While the transplantation of human organs depends on the use of toxic immunosuppressive agents, the use of pig organs will make it possible to, at least in part, alleviate rejection by genetic modification of the animal.

## XENOGRAFT REJECTION

The key obstacle to using pig organs and cells for transplantation in humans has been the strong immune response elicited by porcine antigens. The most immunogenic epitope, the gal-epitope, is generated by the enzyme α-1,3 galactosyl transferase.[2] This epitope is responsible for the hyperacute rejection phenomena. The epitope is expressed on the tissues of all mammals except humans and subhuman primates, which have antibodies against the epitope. If a pig kidney is perfused with human blood, the preformed antibodies react with the gal-epitope. This antibody-antigen reaction elicits an activation of the complement and clotting systems, with subsequent injury to the vascular endothelium and intravascular clotting, a chain of events that results in hyperacute rejection.

**Figure 1:**
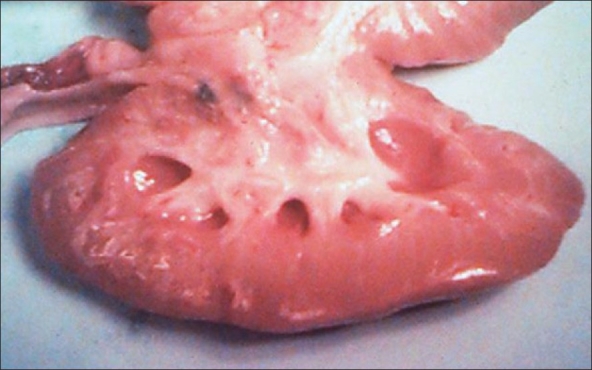
Transgenic pig kidney transplanted to a cynomolgus monkey. The parenchyma is normal 78 days after transplantation (Courtesy of Dr. David White, Robarts Research Institute, London, Ontario, Canada)

In the early 1990s, pigs that were transgenic for a human complement inhibitor, h-DAF, were developed.[3] When the kidneys and hearts from such pigs were transplanted into primates, hyperacute rejection was usually avoided. If the primates were treated with immunosuppressive agents in high doses, the pig organs could, in some animals, function for several weeks or even month [Figure 1].[4–6] However, many of the pig organs were lost early after transplantation due to vascular rejection, while many of the primate recipients died from adverse reactions related to the immunosuppressive treatment. It was concluded that the transgenic pigs constituted a significant advance regarding the avoidance of hyperacute rejection; however, the technology was not ready for clinical trials. Moreover, there was also the potential risk of transmitting porcine endogens retroviruses, (see below). For these reasons, interest in xenotransplantation research abated in the late 1990s.

**Figure 2:**
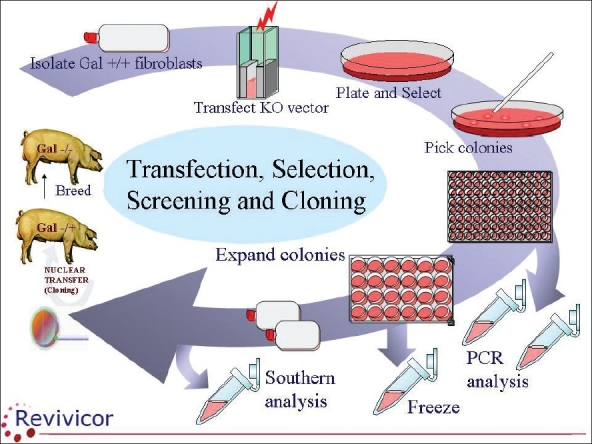
Cartoon showing steps involved in the making of gal-single-knockout pigs. The galactocyltransferase gene has been eliminated on one allele by cloning. Breeding of such pigs will result in gal-double-knockout pigs with the gene being eliminated on both alleles. The tissues of such animals do not express the gal-epitope (Courtesy of Revivicor Inc, Blacksburg, Virginia, USA)

**Figure 3:**
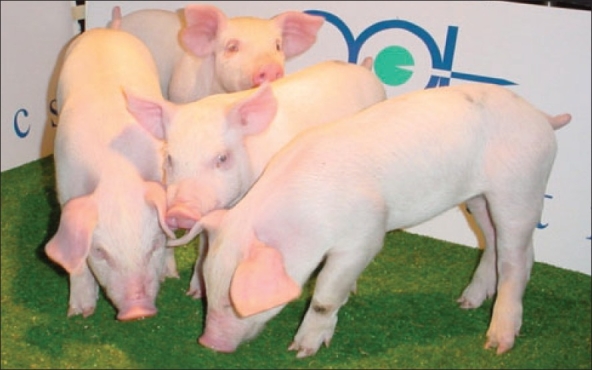
Gal-knockout pigs (Courtesy, Revivicor Inc, Blacksburg, Virginia, USA)

Recently, however, research groups in Boston and Pittsburg succeeded in cloning pigs and in this context, were able to eliminate the gene that encodes α-galactocyltransferase [Figures 2 and 3]. These “gal-knockout pigs” do not express the gal-epitope.[7,8] When kidneys and hearts from such pigs were transplanted into baboons, hyperacute rejection was not encountered and in some immunosuppressed recipients the pig organs survived and functioned for a few months. However, long-term function was prevented by graft rejection, histological examinations revealing thrombotic microangiopathy.[9–11]

Pigs transgenic for human complement regulatory genes and gal-knockout pigs, constitute a significant advance with regards to xenotransplantation, in that the hyperacute rejection has been eliminated and graft survival has been significantly prolonged. Still, all the organs were destroyed by rejection within months. The next step is to combine the two technologies and currently laboratories in the USA and Australia are aiming at making gal-knock out pigs transgenic for h-DAF and for a human gene encoding for an inhibitor of coagulation, (h-TFPI), thereby hopefully alleviating the thrombotic microangiopathy.

## MICROBIOLOGICAL RISKS

With xenotransplantation, there is a risk of transmitting infectious agents from animal to man. With regard to most microbiological agents, the risk can be minimized by using animals from strictly controlled herds. However, such measures will not affect the porcine endogenous retrovirus (PERV). These viruses are a permanent part of the genome in all mammalian species[12] and so all recipients of porcine transplant will inevitably be exposed to PERV. However, the endogenous retroviruses do not replicate or cause disease under physiological conditions. However, in the late 1990s it was found that when pig cells were co-cultured with human cells, the transmission of PERV could occur.[13] Furthermore, the transmission of PERV was found when immune-incompetent mice were injected with porcine cells.[14]

The question was then raised whether the transplantation of porcine tissue into humans might transmit PERV and if the dormant virus in this context might recombine or otherwise be activated in the new environment and thus become pathogenic. If so, the recipient would become infected, as might his/her relatives and other people attending to the patient. In a worst case scenario, the activated viruses would cause an epidemic. The transplantation of organs or cells from pig to man would then be a highly hazardous undertaking.

In the meantime, a number of new technologies for the monitoring of PERV were developed and the application of these tools greatly increased the understanding of the behavior of the virus.[15] When samples were collected from 160 patients who had been treated with various living pig tissues up to 12 years earlier, no evidence of PERV transmission was detected in any patient.[16] However, future patients receiving pig transplants must be carefully monitored for PERV and similar monitoring will have to apply to their families.

## ETHICAL ISSUES

There are several ethical issues pertaining to clinical xenotransplantation. One such issue concerns defining the harm/benefit ratio in the context of the initial clinical attempts. It might also be difficult to institute an appropriate informed-consent procedure. The fact that the patients will have to be monitored for possible infectious diseases for an extended time, possibly for life, is another ethical concern.

Moreover, there are a number of ethical issues with regards to society at large. The public health hazard posed by the possible transmission and activation of PERV created considerable public concern a few years ago. The public debate that took place also generated some emotionally colored skepticism towards xenotransplantation, in which it was argued that “transplantation from animal to man will violate the order of nature”. Other objections were with regard to the safety of the future recipients: “xenotransplantation will constitute experimentation on sick human beings”. It is interesting to note that similar skepticism prevailed in the 1960s and 1970s when human organ transplantation was in its infancy. When it became apparent that transplantation could save a dying patient and bring him back to a normal life, the skepticism and the criticism abated.

The existence of national guidelines and of an official, institutional surveillance system are prerequisites for clinical trials with xenotransplantation. A number of countries, including the USA and the UK, already have guidelines in place, while others are lagging behind. This raises concerns that some such countries might initiate xenotransplantation programs and attract desperate patients in need of a transplant. The WHO has recently issued a statement against such “xenotourism”.

## THE MANY POTENTIAL ADVANTAGES OF XENOTRANSPLANTATION

Once clinical xenotransplantation becomes available there will be no organ shortages and it will be possible to offer transplantation to all patients in need. While patients in many countries currently may have to wait for years for a cadaveric organ, all transplantations can be performed promptly and death on the waiting list will be avoided. Furthermore, with access to pig organs more liberal age limits could be applied, making it possible to accept elderly patients, who are not accepted today.

Eventually, the use of organs from diseased or live human donors will become obsolete. The many legal and practical problems that are associated with the use of organs from deceased donors will become history, a fact that will be of special importance in countries where removing organs from deceased human beings is made difficult by cultural taboos. Moreover, the inevitable ethical problems that accompany the use of related or unrelated living donors, such as coercion and financial arrangements (including organ commerce) will be avoided.

Recently, it has become customary to accept non-optimal human organs for transplantation. With the use of pig organs, all organs will be of optimal quality. Furthermore, it will be possible to keep the warm and cold ischemia times to a minimum.

A further advantage of using pig organs is that the transplant procedure can be prescheduled, allowing for the pretreatment of the recipient with immunosuppressive agents. Such pretreatment has recently become practice at some centers with the aim of facilitating tolerance.

Finally, the use of pig organs would make it possible to alleviate graft rejection by modifying the donor tissue through genetic engineering, thereby making the outcome less dependent on recipient treatment with immunosuppressive agents. Also, there is an important potential immunological pay-off, in that the use of inbred pig strains would provide unlimited access to specific porcine hematopoetic stem cells, cells which could be used in tolerance induction protocols.

Eventually, xenotransplantation has the potential to bring significant cost savings. Thus, the existing organizations for procurement and sharing of organs from deceased donors will become obsolete. The immediate access to pig organs will also result in savings: there will be less need for chronic dialysis treatment and less need for intensive care treatment of patients with end-stage liver, heart and lung disease.

### Pig-to-man organ transplantation

The two prime candidate organs for pig-to-man transplantation are the kidney and the heart and most of the primate studies have focused on these organs. A highly sensitized uremic patient who, for immunological reasons, cannot receive a human kidney and who does not tolerate dialysis could be a potential pig-kidney recipient. If the kidney failed, it could be removed promptly. A patient dying from heart failure could be provided with a pig heart as a bridge until a human heart becomes available. Would it be that the pig heart keeps functioning, it could be retained.

In this regard, there exist some historical experiences. Thus, an attempt to use a pig heart for bridging was performed in the early 1990s and an attempt to use a pig liver for bridging has also been performed. However, both organs failed.[1]

The existence of gene-modified pigs and of new powerful immunosuppressive agents will, however, eventually justify attempts at transplanting pig organs into humans.

### Early trials with pig-to-man cellular transplantation

Some insight into the fate of pig tissue transplanted into humans has been obtained in the context of pilot trials, in which porcine cellular grafts have been transplanted into patients. The transplantation of xenogeneic cells is a simple and safe procedure and rejected cells will, presumably, just fade away. As of today, experience exists with the transplantation of xenogeneic cells in a few patients.

In the early 1990s, a study was performed in Stockholm, Sweden, in which fetal pig islets were injected into diabetic patients.[17] In one patient, who had the islets placed under the kidney capsule of a simultaneously transplanted human kidney, a biopsy taken three weeks after transplantation showed intact porcine cells staining positive for insulin and glucagon. On examination with electron microscopy, viable cells with well-defined secretory granulae were observed.[18] These were the first ever observations of pig cells surviving in the human body.

In the late 1990s in Boston, USA, a few patients with Parkinson's disease had fetal pig mesencephalic cells injected into their brains. One of the patients died suddenly of pulmonary embolism seven months after the transplantation. Examination of the patient's brain revealed three surviving clusters of viable pig cells. The pig neurons had extended axons into the patient's brain. This was the first documentation of the neural pig cells surviving in the human brain.[19]
